# A counting method of whiteflies on crop leave images captured by AR glasses based on segmentation and improved YOLOv11 models

**DOI:** 10.3389/fpls.2025.1687282

**Published:** 2025-10-09

**Authors:** Shilong Zhao, Jun Lyu, Shuhua Liu, Zelin Feng, Heping Ling, Jiabao Jiao, Zhaoxin Ni, Baojun Yang, Qing Yao, Ju Luo

**Affiliations:** ^1^ School of Computer Science and Technology (School of Artificial Intelligence), Zhejiang Sci-Tech University, Hangzhou, China; ^2^ School of Information Science and Engineering, Zhejiang Sci-Tech University, Hangzhou, China; ^3^ State Key Laboratory of Rice Biology, China National Rice Research Institute, Hangzhou, China; ^4^ School of Information and Control, Keyi College of Zhejiang Sci-Tech University, Shaoxing, China; ^5^ Jinhe Nengyuan Technology Co., Ltd., Changzhou, China

**Keywords:** whitefly, AR glasses, image segmentation, object detection, automatic counting

## Abstract

The whitefly (*Bemisia tabaci*) is a globally distributed agricultural pest. While accurate monitoring of this species is crucial for early warning systems and efficient pest control, traditional manual monitoring methods suffer from subjectivity, low accuracy with large populations, and arduous data traceability. To surmount these challenges, this paper proposes an automatic counting method for whitefly adults and late-instar nymphs, based on whitefly images acquired using augmented reality (AR) glasses and a segmentation-then-detection approach. Acquired by the surveyors wearing AR glasses, the images of whiteflies on the undersides of crop leaves are transmitted to a server via Wi-Fi/5G. The system enables the automatic whitefly counting model to enumerate the adult and late-instar nymph populations, and the results can be viewed on both the AR glasses and mobile devices. The study utilizes Mask2Former-Leaf to segment the foreground primary leaf in pursuit of the minimal influence of non-primary leaf areas and background noise in the images, and detects tiny whitefly adults and late-instar nymphs in high-resolution images by involving the YOLOv11-Whitefly detection model. This model integrates Slicing Aided Hyper Inference (SAHI) capability, and can enormously amplify the feature representation of tiny objects by slicing large images through overlapping windows for both training and inference. Furthermore, DyCM-C3K2 is introduced into the YOLOv11 backbone network, which enhances the detection capability for small whitefly targets by dynamically generating input-dependent convolutional kernels, and injecting global contextual information into local convolution operations. Also, a Multi-Branch Channel Re-Weighted Feature Pyramid Network (MCRFPN) is designed to replace the original neck network, optimizing the fusion between superficial and deep features. In contrast to mainstream detection models such as YOLO, RTMDet, Cascade-CNN, DETR, and DINO, YOLOv11-Whitefly demonstrates superior performance—attaining an average recall rate of 86.20%, an average precision of 84.25%, and an mAP_50_ of 91.60% for whitefly adults and late-instar nymphs. With the purpose of visualizing the whitefly infestation data, this paper developed an intelligent whitefly survey system that provides on-site visualization of whitefly images integrated with their adult and late-instar nymph counting results. This facilitates surveyors in understanding pest populations and formulating scientific control decisions.

## 1 Introduction

The whitefly (*Bemisia tabaci*) refers to a globally distributed agricultural pest incurring substantial economic losses in agriculture. It poses formidable challenges to integrated pest management based on the following factors: a broad host range, the habit of damaging the undersides of crop leaves, diverse transmission pathways, rapid generation turnover, and strong pesticide resistance (Chu and Zhang, 2018). Timely and accurate assessment of whitefly population density in the field is a prerequisite for efficient pest control. According to the national technical standard NY/T 2950-2016 (2016) in China, whitefly nymphs are classified into two groups: low-instar nymphs (1st and 2nd instars) and late-instar nymphs (3rd, 4th, and pseudo-pupae). These standards dictate that only adults and late-instar nymphs are to be used as survey targets and statistical metrics. Currently, two primary methods are employed for whitefly surveys. The first is manual field surveying, where investigators physically enter fields, quickly turn over leaves, and visually estimate and manually record the number of adult and late-instar nymph whiteflies on the leaf’s underside. The second is the yellow sticky trap method, which involves using traps to attract and manually count flying adults. However, due to the tiny size of the insect, both manual identification and counting methods endure inefficient survey, myriad estimation errors (especially during peak periods), and impedance in data traceability.

At the current stage, automatic counting of tiny insect pests using yellow sticky traps is emerging as a principal research direction for bolstering the intelligence and accuracy of pest monitoring. Traditional machine vision methods for pest counting primarily lean on handcrafted features combined with machine learning classifiers for identification. For instance, (Deqin et al., 2018) developed a whitefly counting method for vegetable pests by engaging a structured random forest-based image segmentation algorithm, an irregular structural feature extraction algorithm, and sub-algorithms for interference target removal and detection. Similarly, (Zhong et al., 2018) leveraged manually extracted global features such as shape, texture, and color, in conjunction with HOG local features and Support Vector Machines. The target was to identify and classify images of yellow sticky traps containing six types of pests, including whiteflies and thrips. Despite their abilities to guarantee high recognition accuracy under specific conditions, the reliance of these methods on manual feature engineering suppresses their adaptability to intricate and variable field circumstances, resulting in poor capabilities of generalization. Thanks to the successful application of deep learning in image recognition and object detection, researchers commence with employing improved object detection models for pest identification and counting on yellow sticky traps. For example, to detect whiteflies and thrips on yellow sticky traps, (Li et al., 2021) proposed the TPest-RCNN model, marking an improvement based on Faster R-CNN. (Bai et al., 2024) put forth a multi-insect recognition framework termed MS-P2P in light of point regression by integrating YOLOv7-tiny, LAHead, and the Hungarian matching algorithm. The average F1 score for whitefly detection in their self-built cotton field yellow sticky board dataset was 80.9%. Nevertheless, the yellow sticky trap method attracts merely flying pests and is non-applicable to surveying the number of nymphs. Besides, during periods of high pest populations, the saturated sticky surface exacerbates counting accuracy, and the sticky traps compel regular replacement.

Although methods based on yellow sticky traps and computer vision have accomplished progress in the automatic counting of specific flying pests, an immense portion of pest surveys still entail manual field surveys. (de Castro Pereira et al., 2022) presented an improved YOLOv4 deep learning strategy for images of soybean leaf whiteflies captured in a laboratory setting. The strategy starts with training the model weights using cropped images, followed by image stitching to merge detection results. The model harvested an average F1 score of 0.87 for detecting whiteflies of different instars. Unfortunately, the dataset in the paper was collected under controlled laboratory conditions using single leaf images, and the model’s performance in natural field conditions still needs validation. (Bereciartua-Pérez et al., 2022) described a density map estimation method for counting whiteflies on eggplant leaves collected from Spanish greenhouses. They formulated a fully FCRN and a tailored GSP strategy to plot Gaussian density maps for precise insect localization. According to experiments, this method achieved a coefficient of determination R² of 0.97 for whitefly counting, and the model was deployed to a mobile application, providing an efficient tool for precise pest management. (Feng et al., 2024) collected whitefly images on cotton leaves. To modify a YOLOv8s-based model, they replaced its C2F module with a Swin-Transformer, then introduced a P2 branch structure in the detection head, and carried out Slicing Aided Hyper Inference (SAHI) for image preprocessing. This augmented model derived a mAP_50_ of 92.00% for whitefly identification, and was ultimately integrated into a Raspberry Pi edge computing terminal, offering a feasible solution for real-time field pest monitoring. Most of the aforementioned methods construct counting models based on whitefly adults on single crop leaves photographed in controlled or relatively simple scenarios. These approaches confront a confined image background that hinders the models from performing whitefly detection tasks on various crop leaves in convoluted field environments—not to mention they are normally unable to detect smaller whitefly late-instar nymphs.

To address the low efficiency and high error rates of manual field surveys, as well as the inability of yellow sticky traps to monitor nymphs, this paper proposes an automatic whitefly counting method based on augmented reality (AR) glasses and a segmentation-then-detection approach. The system leverages the advantages of AR glasses, including hands-free operation, real-time visual display, and voice control functionalities, which enables surveyors to efficiently and conveniently acquire high-resolution whitefly images in complex field environments. To prevent false positives from non-primary leaves and the influence of complex backgrounds, we utilize Mask2Former-Leaf to achieve the precise extraction of foreground primary leaves. Moreover, to achieve high-precision detection of tiny whitefly adults and late-instar nymphs in images, we designed the YOLOv11-Whitefly detection model. This model integrates SAHI capability to amplify the feature representation of tiny whiteflies by slicing large images through overlapping windows. Furthermore, the DyCM-C3K2 module and MCRFPN are introduced to enhance the model’s detection capabilities for small whitefly targets. Finally, we developed an intelligent whitefly survey system based on these core technologies. This system realizes efficient image acquisition, high-precision whitefly detection and counting, on-site result display, and data traceability. It ultimately provides surveyors with valuable insights into pest populations, thereby facilitating the formulation of scientific control decisions.

## 2 Materials and methods

### 2.1 Intelligent monitoring and survey method for whitefly

**Figure 1:**
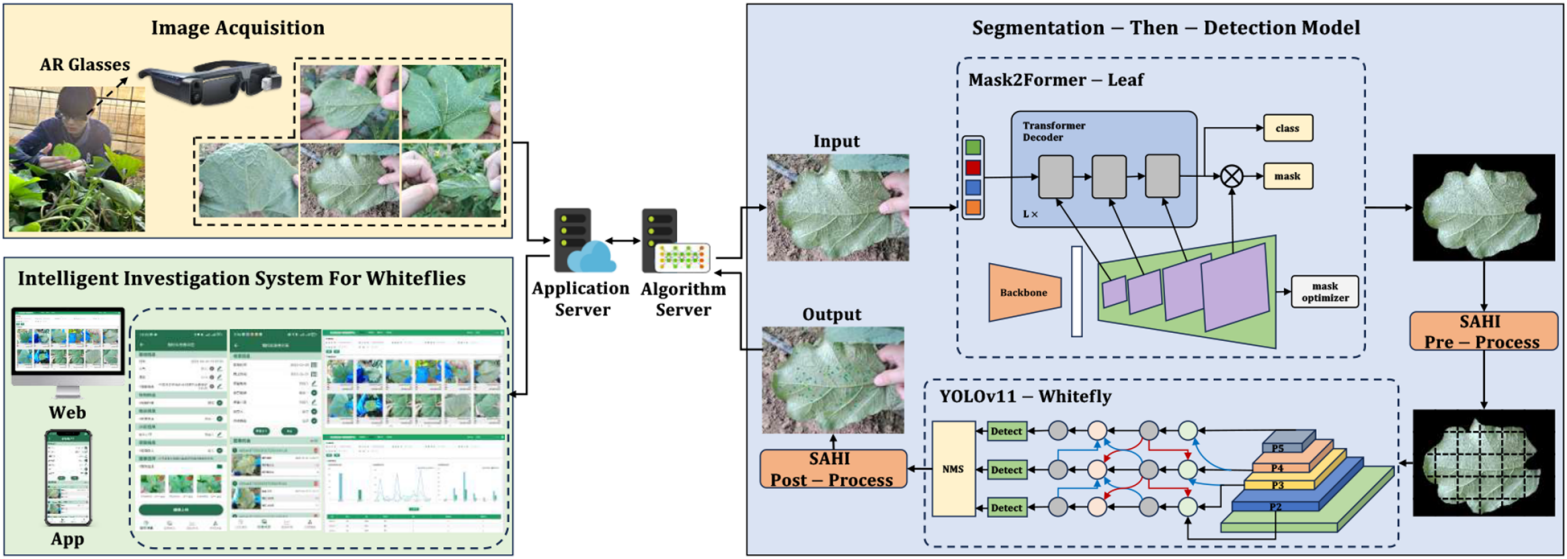
Intelligent monitoring and survey method for whitefly.

With the intent of enhancing the intelligence and precision of whitefly monitoring and surveying, surveyors wear AR glasses to quickly capture high-definition whitefly images via voice control. These images are then transmitted to a server via Wi-Fi/5G, which activates a segmentation-then-detection model to detect the whitefly adults and late-instar nymphs in the images. Finally, the detection and counting results are transmitted back and displayed on the AR glasses as well as the Web and APP terminals of the intelligent survey system for visualization and subsequent data management. The architecture of the intelligent survey method for whitefly monitoring and surveying is illustrated in **Figure 1**.

### 2.2 Image acquisition and dataset construction

This study selected the SUPERHEXA AR glasses from Superhexa Century Technology as the image acquisition device, intending to precisely capture high-definition images of tiny whiteflies on the undersides of crop leaves in the field and adapt to varying outdoor lighting conditions. The device features a 50-megapixel high-definition camera, a 3000-nits peak brightness optical display, and fast autofocus capabilities, ensuring image quality in intricate environments. Furthermore, its voice control function notably underpins the collection efficiency during field operations.

**Figure 2:**
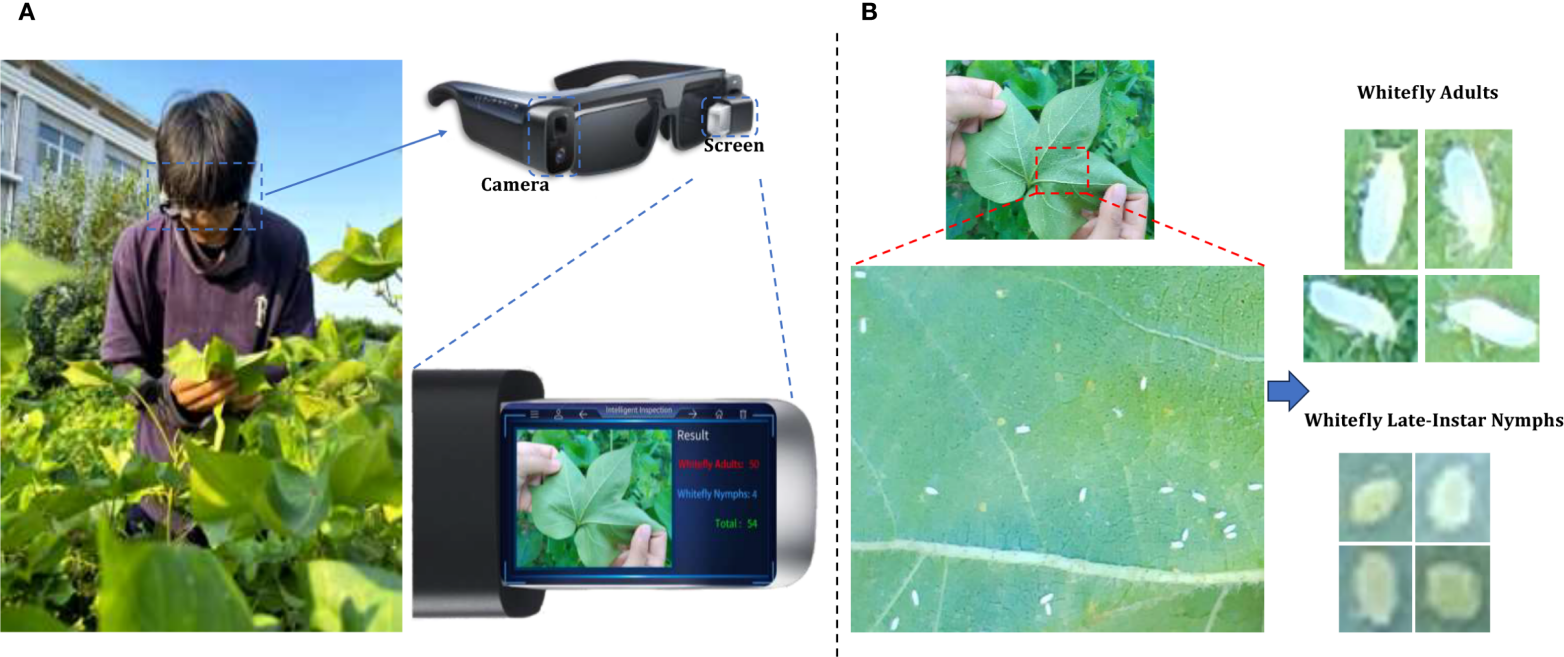
An example of whitefly image acquisition. **(A)** Surveyors wearing AR glasses and capturing whitefly images; **(B)** Image of adult and late-instar nymph whiteflies on leaf underside.

During the high-incidence seasons for whiteflies from May to October 2023 and May to October 2024, image collection was conducted in strict accordance with whitefly survey standards at the Jiangsu Academy of Agricultural Sciences and the Jinan Vegetable Research Science and Technology Park in Shandong Province. As depicted in **Figure 2**, with AR glasses, surveyors captured images using voice commands, while gently flipping and flattening the leaves with both hands. Across various times of day and weather conditions, a total of 5124 images of whiteflies were finally garnered on the undersides of five different crops (i.e. pepper, cotton, cucumber, eggplant and tomato), all of which were 4080×3072 pixels and included the whiteflies in both adult and late-instar nymph stages. The detailed data information is listed in **Table 1**. The per-image counts for these pest stages were characterized by a mean of 15.50 for adults and 5.46 for late-instar nymphs, with corresponding standard deviations of 29.15 and 24.31. The number of adults ranged from 0 to 380 per image, while the number of late-instar nymphs varied from 0 to 514. This wide range in pest density, including instances of extremely low and high populations, reflects the diverse and complex nature of whitefly distribution in field environments.

**Table 1:** Whitefly pest information on the undersides of five crop leaves.

Crop species	Images	Number of images	Number of whiteflies	
			Adult	Late-instar nymph
Pepper	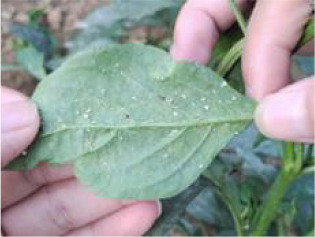	1907	15487	12096
Cotton	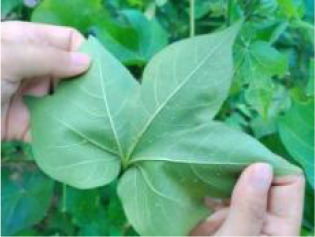	1057	23353	1964
Cucumber	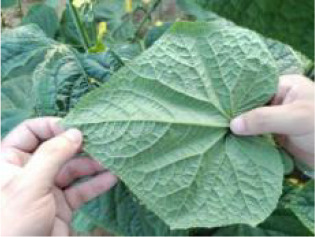	856	16856	5060
Eggplant	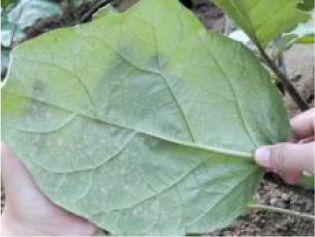	617	19888	8099
Tomato	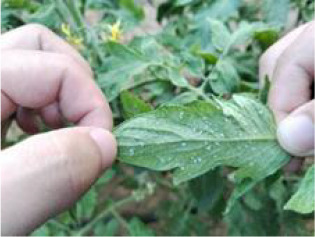	687	3858	785

To reduce interference from complex field backgrounds and non-primary leaf whiteflies, accurate extraction is essential for the foreground primary leaf. First, we randomly sampled a portion of images from each of the five collected crops. We then used the SAM (Kirillov et al., 2023) for auxiliary annotation and performed manual correction with the Labelme tool. The annotated data was saved in JSON format, and subsequently used to construct a primary leaf semantic segmentation dataset. Subsequently, for the whitefly object detection task, we used the LabelImg tool to annotate the categories and location information of whitefly adults and late-instar nymphs on all image data from the five crops, saving the data as XML files. Both the segmentation and detection datasets were then split into training, validation, and test sets with an 8:1:1 ratio, respectively.

### 2.3 Whitefly counting model

In natural field environments, images of whiteflies captured using AR glasses often contain more than a single leaf, so directly applying a detection model to the entire image would detect and count the whiteflies on other non-primary leaves. This contradicts the standard on agricultural whitefly pest forecasting, which specifies that the pests should be quantified on a single leaf. To address the flaw, this paper proposes a segmentation-then-detection whitefly automatic counting method. More precisely, the Mask2Former-Leaf foreground primary leaf segmentation model was established in the first stage, which extracts the foreground primary leaf from the image, and discards other non-primary leaves and irrelevant backgrounds to prevent false positive whitefly detection. The second stage relies on an object detection method to create the YOLOv11-Whitefly detection model that detects and counts the adult and late-instar nymph whiteflies specifically on the segmented foreground primary leaf.

#### 2.3.1 Mask2Former-leaf model

In this study, semantic segmentation is employed as a prerequisite step for our core task of whitefly detection, specifically for precisely extracting the foreground primary leaf region in convoluted field environments. The model classifies image pixels as primary leaf or background, filtering out non-primary leaves and irrelevant background interference, thus providing premium target input images for subsequent detection. Mainstream semantic segmentation models were involved for experimentation in conjunction with post-processing optimization to achieve foreground primary leaf segmentation at minimal cost.

**Figure 3:**
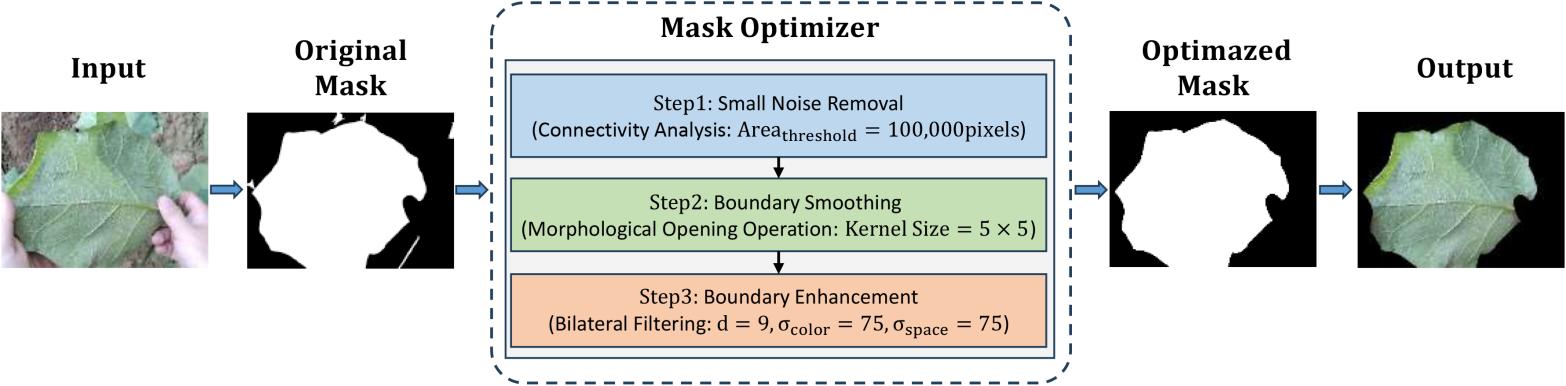
Structure of the foreground primary leaf mask post-processing optimizer.

To build our foreground primary leaf segmentation model, the study ultimately chose Mask2Former (Cheng et al., 2022). Its highly versatile architecture, which leverages a mask attention mechanism and an efficient multi-scale feature fusion strategy, is particularly well-suited for our segmentation task. To further reinforce the foreground primary leaf segmentation masks, a mask post-processing optimizer was developed that encompasses connected component analysis, morphological operations, and edge enhancement techniques to alleviate noise and rough boundaries. This three-stage process commences by performing connected component analysis to filter out small noisy regions based on a predefined area threshold, so that the main leaf body can retained; afterwards, it applies a morphological opening operation with a fixed-size structural element to smooth mask edges, aimed at removing irregularities and filling small holes for a more regular contour; finally, bilateral filtering is used to internally smooth non-edge regions and enormously raise edge clarity between the primary leaf and background. This yields a highly refined segmentation with accurate boundaries, as depicted in **Figure 3**.

#### 2.3.2 YOLOv11-whitefly model

YOLOv11 (Khanam and Hussain, 2024), the latest iteration in the YOLO series, marks a number of innovations across its model architecture, feature fusion, and computational efficiency. It incorporates the C3K2 module that combines depth-wise separable convolutions with residual connections, relieving computational load, and optimizing small object detection; the SPFF module dynamically weights and fuses multi-scale features, dramatically strengthening feature extraction capabilities in complicated scenarios; and the dynamic inference strategy adaptively chooses pathways based on image complexity, fostering a balance between inference speed and accuracy.

**Figure 4:**
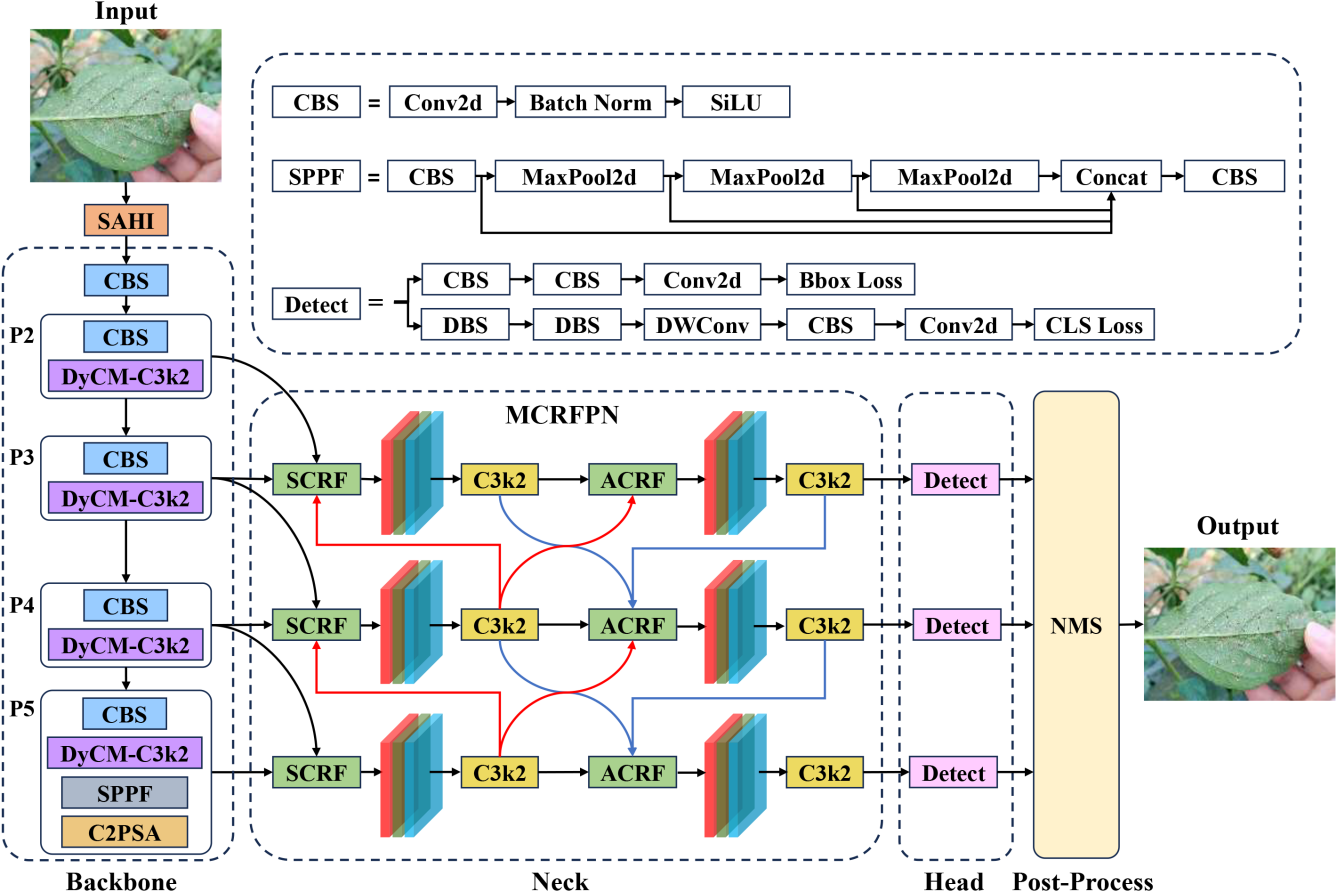
The structure of the YOLOv11-Whitefly model.

To better suit the whitefly detection task, this study tuned the YOLOv11s object detection model to launch the YOLOv11-Whitefly detection model. First, whiteflies are less than 1 mm in length, and the images in the dataset are all 4080×3072 pixels. This implies an extremely small proportion of pixels occupied by the whitefly adults and late-instar nymphs, incurring insufficiency in feature information. To conquer this problem, SAHI was integrated into both the training and inference stages of YOLOv11. The method divides the large images into overlapping sub-images using a sliding window, performs inference on each sub-image, and combines the results to obtain the final detection for the original high-resolution image. Second, given the dense and often overlapping distribution of whiteflies on leaves, coupled with interference from non-target pests such as dead whiteflies, DyCM-Conv was proposed to intensify YOLOv11’s backbone network by modifying its C3K2 module. Such a module dynamically generates convolution kernels adapting to input features, significantly underpinning the model’s ability to extract traits from tiny whiteflies. Its context-aware mixing mechanism captures long-range dependencies within images, boosting detection robustness for dense and overlapping targets, while also suppressing interference from elements like dead insects. Finally, because whitefly sizes vary drastically across images of different crop leaves, this study introduced the MCRFPN to replace the original neck network, which tailors the fusion of different scale features through its SCRF and ACRF modules, mitigating the issue of insufficient receptive fields for small objects and reinforcing the model’s multi-scale feature extraction and fusion capabilities. The structure of the YOLOv11-Whitefly model is visualized in **Figure 4**.

##### 2.3.2.1 SAHI

**Figure 5:**
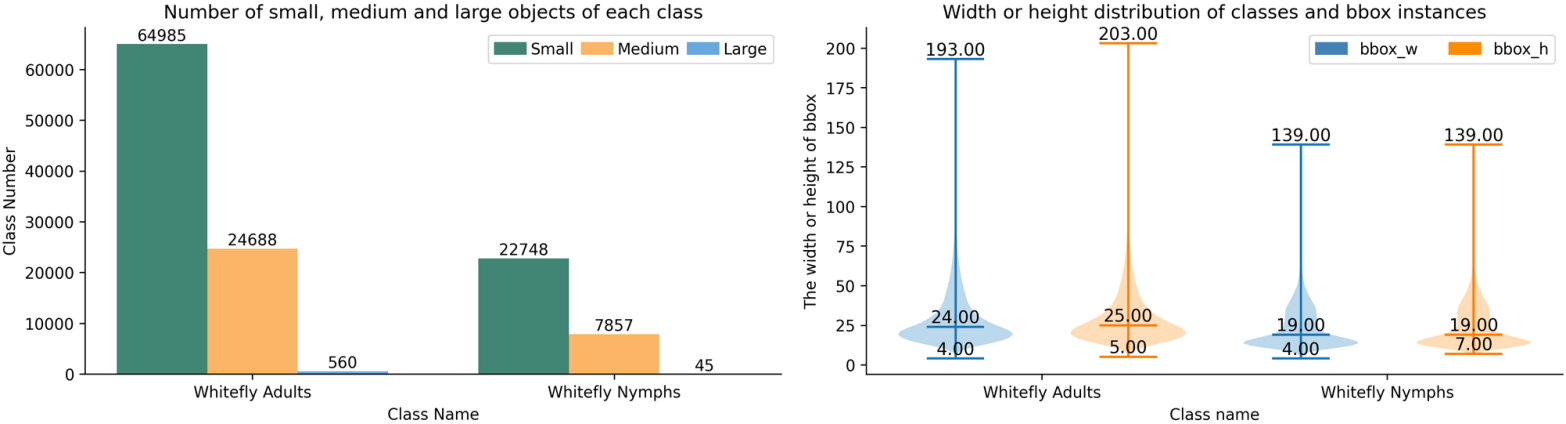
Distribution of object scale counts and aspect ratios for each class.

As per the standard on MS COCO classification, objects are categorized by absolute pixel area into the small objects less than 32×32 pixels, the medium objects between 32×32 and 96x96 pixels, and the large objects greater than 96×96 pixels. A statistical analysis of the whitefly dataset, based on these criteria, reveals that the median width and height are 24×25 pixels for whitefly adults, and 19×19 pixels for late-instar nymphs. Critically, small objects account for 72.01% and 74.21% of adult and late-instar nymphs, respectively. Given a resolution of 4080×3072 pixels for all the images, this dataset is characteristic of a high-resolution, small object detection task. The distribution of object scales and width-height distributions for each category is shown in **Figure 5**.

SAHI (Akyon et al., 2022) is an enhancement strategy specifically designed for high-resolution small object detection, whose core philosophy is to divide large images into smaller sub-images via overlapping sliding windows. Each sub-image is then independently analyzed, with the results merged. This process intends to upgrade the model’s sensitivity to small targets by expanding the relative size of objects within each sub-image. The execution flow is as follows:

1. Image slicing: The input image is decomposed into multiple sub-images of fixed size S×S. To preclude edge targets from being cropped, adjacent sub-images retain a certain overlap area, with the overlap ratio controlled by an overlap ratio α.
2. Sub-image detection: Each sub-image undergoes independent inference through the detection model. For every sub-image, a set of local object detection bounding boxes, class labels, and confidence scores are generated.
3. Result merging: After mapping the sub-image detection boxes from their local coordinates to the global coordinates of the original image, the application of NMS ensues to eliminate redundant detection boxes originated from the same target in overlapping areas, while preserving the prediction results with the highest confidence.

**Figure 6:**
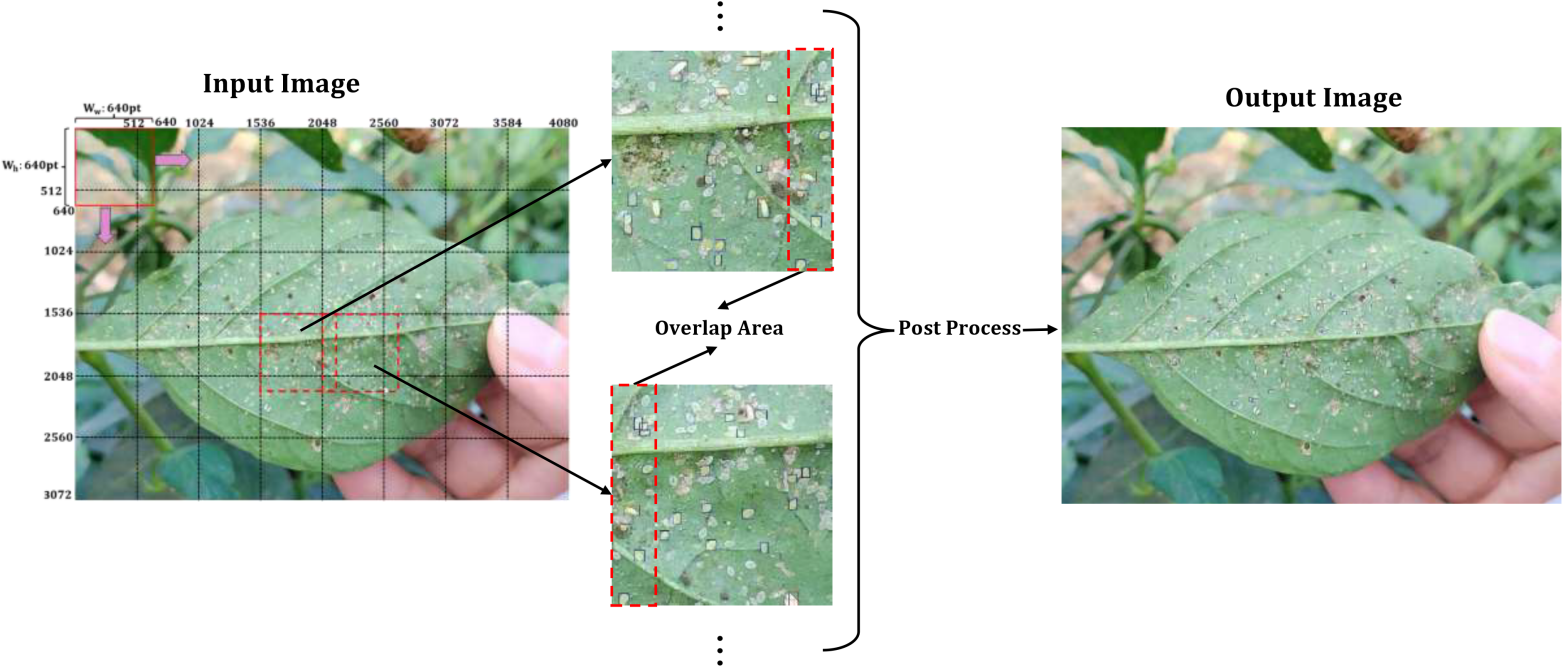
SAHI workflow in the whitefly dataset.

SAHI employs a divide and conquer strategy to transform tiny objects in high-resolution images into prominent targets in sub-images, which evidently mitigates the issue of feature loss provoked by extremely small object sizes. In this study, the sub-image size is 640×640 pixels, with an overlap rate α of 0.2. During the phase of model training, images were sliced according to these parameters, and any sub-images without targets were discarded as invalid. For the inference phase, the same parameters were adopted to slice the sub-images—each detected individually, and the eventual detection outcomes were obtained by merging with NMS. The workflow is shown in **Figure 6**.

##### 2.3.2.2 DyCM-C3K2

**Figure 7:**
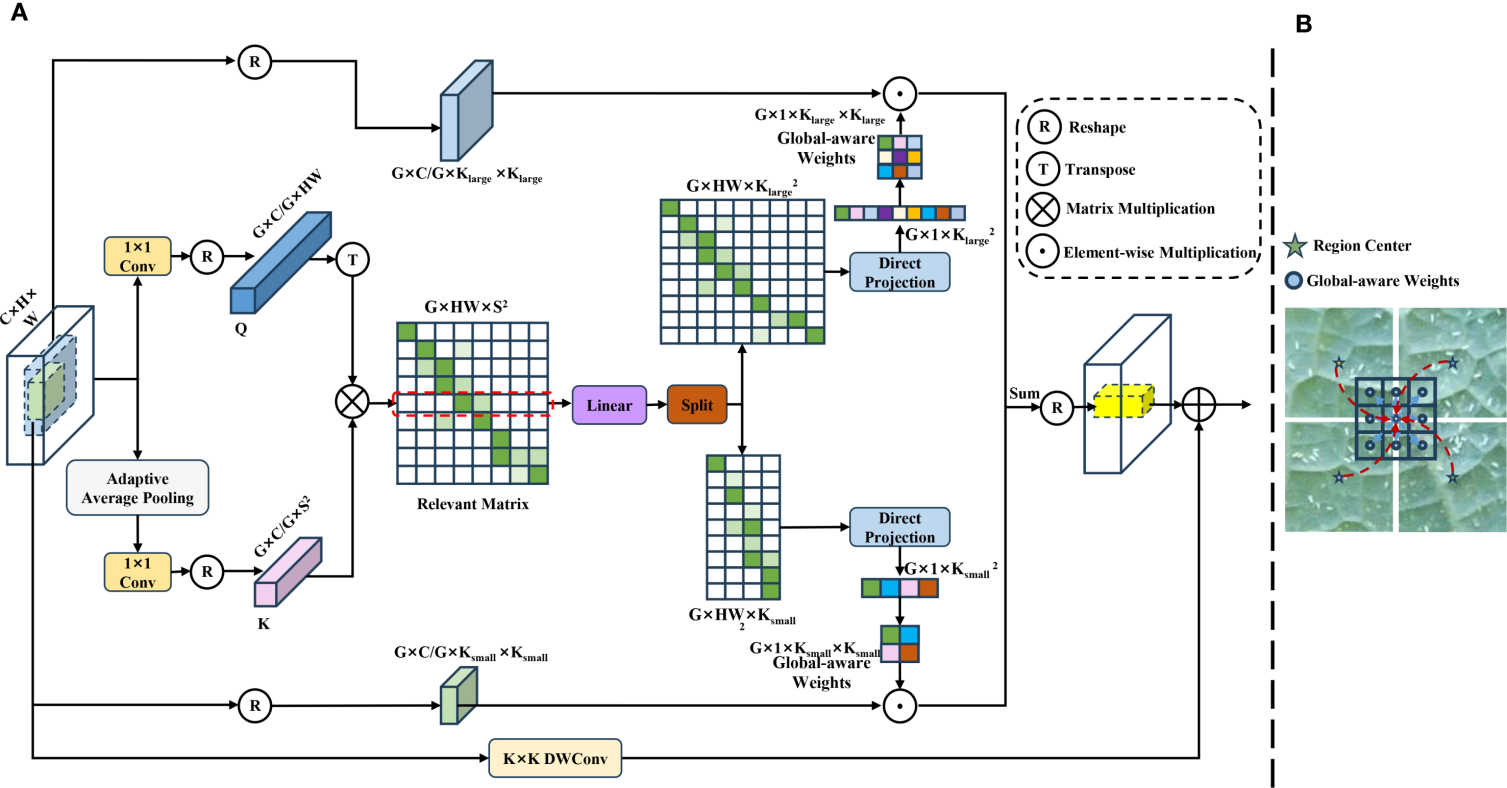
**(A)** A schematic diagram of DyCM-Conv; **(B)** DyCM-Conv’s ability to capture long-range dependencies and maintain inductive bias.

Confronting the drawbacks of the C3K2 module in conventional YOLOv11, such as poor adaptability of static convolution kernels, confined receptive fields, and paucity of feature selectivity, this paper enhances the backbone network by replacing the standard convolutional layer in the bottleneck of the C3K2 module with Dynamic Context-Mixed Convolution (DyCM-Conv). While retaining the cross-stage residual connections, DyCM-Conv dynamically gives rise to convolution kernels, allowing the model to fuse multi-scale contextual information and model long-range dependencies while preserving the inductive bias of traditional convolutions. **Figure 7** unveils its structure and how it captures long-range dependencies during the convolution process. Its essential concept lies in computing the correlation between each spatial position of a feature map and multiple global region centers, which engenders a relevance matrix that designates the strength of inter-position correlations. The matrix is then learnably transformed into a dynamic convolution kernel, injecting global contextual information directly into the convolution kernel. The key implementation steps are elucidated as follows:

1. Formation of the Relevance Matrix: In computer vision tasks, tokens are often generalized as feature units with independent semantic or contextual associations. Given an input feature map $X=R^{C\times H\times W}$ , each spatial point is treated as a token. A 1x1 convolution spawns a query (Q) that preserves spatial information. Concurrently, S×S region center adaptive average pooling and another 1x1 convolution create a key (K) that aggregates global information, followed by the channel-wise division of both Q and K into G groups. Within each group, a matrix multiplication is performed to compute a relevance matrix $R_{g}=Q_{g}^{T}K_{g}\in R^{HW\times SS}$ , where the i-th row indicates the correlation between the i-th pixel and all region centers.
2. Generation of the Dynamic Convolution Kernels: Each token’s relevance matrix row is processed by a linear layer to integrate weights from an array of region centers. This output is then split to represent the properties of predefined $K_{large}$ and $K_{small}$ convolution kernels. Thereafter, relevance values are aggregated and normalized by a direct projection which includes a learnable linear layer and a softmax function. In the end, these weighted rows are reshaped into dynamic convolution kernels of sizes $K_{large}\times K_{large}$ and $K_{small}\times K_{small}$ .
3. Context-Mixed Dynamic Convolution Workflow: This module operates by virtue of a grouped parallel processing approach. Each group arouses one large and one small dynamic convolution kernels, which are both shared across the channels within that group. Afterwards, the generated large and small kernels perform convolution operations on the input feature map independently. As a result, another branch processes the same original input feature map using a DWConv. Finally, the module’s final output is formed by merging the output feature maps from the large dynamic convolution kernel branch, the small dynamic convolution kernel branch, and the DWConv branch.

##### 2.3.2.3 MCRFPN

**Figure 8:**
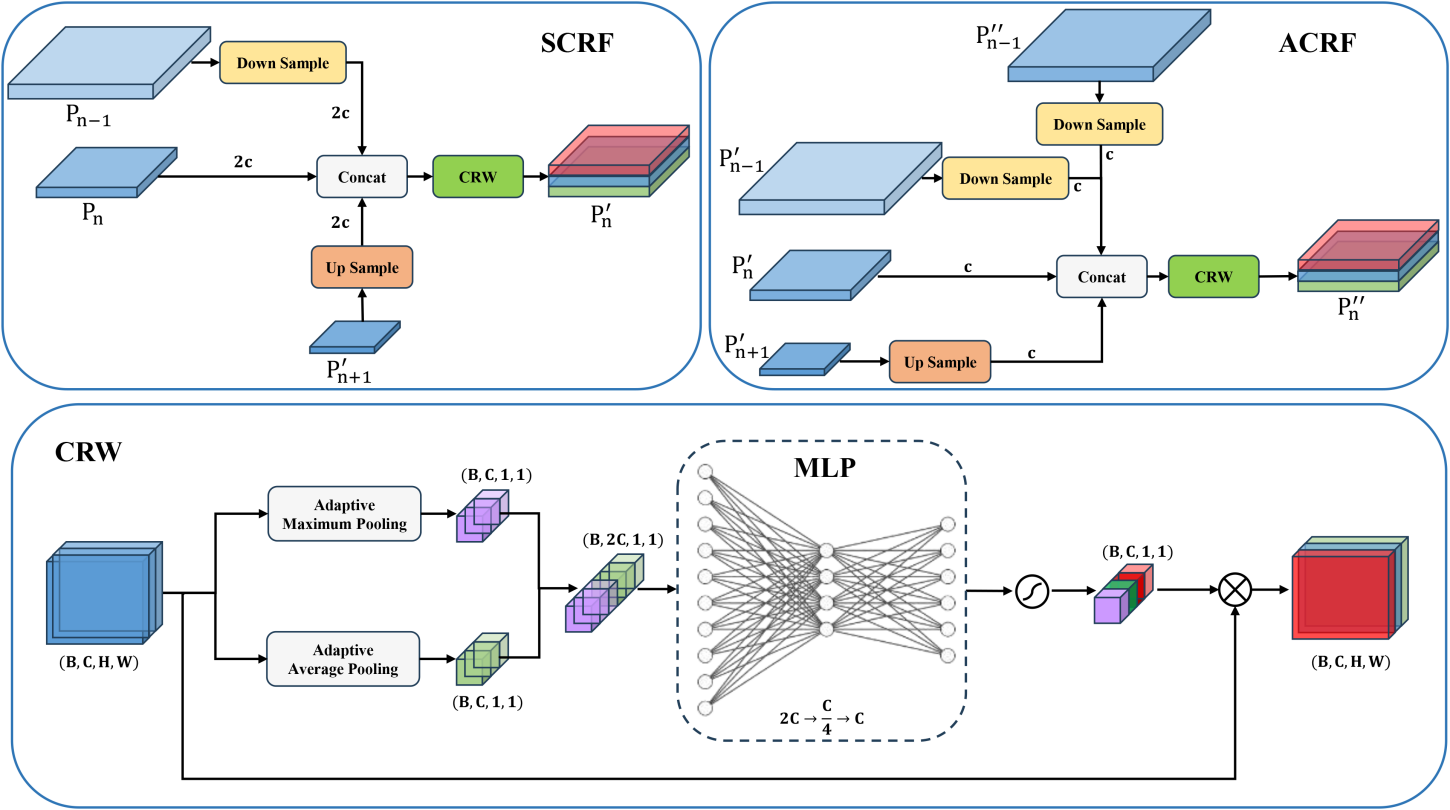
SCRF, ACRF, and CRW structures.

YOLOv11 enhances multi-scale feature fusion via top-down and bottom-up pathways. However, its original neck network’s focus on high-level semantic features and simple, fixed-weight fusion mechanism often leads to insufficient integration of high-resolution features from superficial layers, causing a significant loss of crucial details for small objects. To address this, this paper proposes Multi-Branch Channel Re-Weighted Feature Pyramid Network (MCRFPN) as an alternative to the original neck network. The MCRFPN architecture can be seen in the neck portion of the YOLOv11-Whitefly model in **Figure 4**. The MCRFPN merges the superficial re-weighted fusion (SCRF) and the advanced re-weighted fusion (ACRF), and embeds the channel re-weighted (CRW) strategy to optimize feature fusion. **Figure 8** shows the structures of SCRF, ACRF, and CRW.

The SCRF module is embedded at the superficial connection point between the backbone and neck of the network. It enhances feature representation by fusing outputs from the same level of the backbone $P_{n}$ with superficial feature outputs $P_{n-1}$ and deeper outputs from the neck $P_{n+1}^{'}$ . Embedded deeper within the Neck, the ACRF module improves multi-scale gradient information interaction by fusing superficial $P_{n-1}^{'}$ , mid-level $P_{n}^{'}$ , deep $P_{n+1}^{'}$ from the earlier part of the neck and superficial feature output $P_{n-1}^{''}$ from the end of the neck. These operations of SCRF and ACRF are expressed in (Equations 1 and 2).


(1) $$\begin{array}{c} P_{n}^{‘}=CRW\left(Concat\left(Down\left(P_{n-1}\right),\;P_{n},\;Up\left(P_{n+1}^{'}\right)\right)\right) \end{array}$$

(2) $$\begin{array}{c} P_{n}^{''}=CRW\left(Concat\left(Down\left(P_{n-1}^{'}\right),\;P_{n}^{'},\;Up\left(P_{n+1}^{'}\right),\;Down\left(P_{n-1}^{''}\right)\right)\right) \end{array}$$

Here, $P_{n}$ , $P_{n}^{'}$ and $P_{n}^{''}$ represent the *n* level feature outputs from the backbone network, the first half of the neck, and the second half of the neck, respectively; Up(·) signifies an upsampling operation; Down(·) denotes a downsampling operation; Concat(·) refers to concatenation along the channel dimension; and CRW(·) serves as a channel re-weighting strategy.

In a feature map with a few parameters, Channel Re-Weighting (CRW) is a feature fusion optimization method to adaptively adjust the importance of different channels, with the purpose of boosting the semantic representation capabilities of multi-scale feature fusion. To commence its implementation, GMP and GAP are used on the input feature map to capture channel-level global contextual information. The pooled results are then concatenated and fed into a lightweight fully connected network. Including two fully connected layers and an activation function layer, the network outputs a normalized weight vector that quantifies each channel’s semantic importance. Finally, this weight vector is multiplied element-wise with the original feature map to enhance the contribution of crucial channels. These operations of CRW are expressed in (Equation 3).


(3) $$\begin{array}{c} X_{out}=X_{in}⊗\left(\sigma \left(W_{2}\cdot \delta \left(W_{1}\cdot Concat\left(GMP\left(X_{in}\right),\;GAP\left(X_{in}\right)\right)\right)\right)\right) \end{array}$$

Here, $X_{in}$ means the input feature map; $X_{out}$ denotes the output feature map; GMP(·) and GAP(·) represent global max pooling and global average pooling respectively; $W_{1}\in R^{\frac{c}{4}\times 2c}$ and $W_{2}\in R^{c\times \frac{c}{4}}$ are learnable parameters; δ signifies an ReLU activation function; and σ refers to a Sigmoid function.

### 2.4 Experimental configuration

The computational framework for all experiments in this study was built on an Intel Core i9–13900 processor with a base frequency of 3.0 GHz, 128 GB of RAM, and two NVIDIA GeForce RTX 4090 GPUs. The software environment comprised the Ubuntu 24.04.2 operating system, PyTorch 2.2.0 as the deep learning framework, CUDA 11.8, and Python 3.8.17.

The semantic segmentation models were trained for 50,000 iterations with a batch size of 4, and an input image size of 2048 x 1536 pixels. The target detection models were trained for 200 epochs with momentum set to 0.937, weight decay set to 0.0005, and a batch size of 16. The initial learning rate was 0.01, using the SGD algorithm. All models were trained from scratch on our custom dataset without the use of pretrained weights from external sources.

### 2.5 Performance evaluation

To provide a robust and objective evaluation of our foreground primary leaf segmentation model’s performance, we utilize a comprehensive set of widely-adopted metrics in semantic segmentation. These metrics include Pixel Accuracy (PA), Intersection over Union (IoU), and the Dice coefficient (F1-score), with their formulas defined as in (Equations 4-6), respectively.


(4) $$\begin{array}{c} PA=\frac{TP+TN}{TP+TN+FP+FN} \end{array}$$

(5) $$\begin{array}{c} IoU=\frac{\left|A\cap B\right|}{\left|A\cup B\right|} \end{array}$$

(6) $$\begin{array}{c} F_{1}=\frac{2\left|A\cap B\right|}{\left|A\right|+\left|B\right|} \end{array}$$

Pixel Accuracy (PA) provides a fundamental measure of overall classification performance by calculating the ratio of correctly classified pixels to the total number of pixels. The IoU measures the accuracy of the segmentation algorithm by calculating the ratio of the intersection area between the ground truth and the segmentation result to their union area. When the F1-score assesses the segmentation precision of the algorithm, the area of overlap between the segmentation result and the ground truth are computed twice, then divided by the sum of their individual areas.

With the target of objectively evaluating the detection performance of the whitefly detection model, Precision (P), Recall (R), Average Precision (AP), and mean Average Precision (mAP) were engaged as evaluation indicators, and the formulas are expressed as (Equations 7-10), respectively.


(7) $$\begin{array}{c} P_{k}=\frac{TP_{k}}{TP_{k}+FP_{k}} \end{array}$$

(8) $$\begin{array}{c} R_{k}=\frac{TP_{k}}{TP_{k}+FN_{k}} \end{array}$$

(9) $$\begin{array}{c} AP_{k}=\int_{0}^{1}P_{k}\left(R_{k}\right)dR_{k} \end{array}$$

(10) $$\begin{array}{c} mAP=\frac{\sum_{k=1}^{n}AP_{k}}{n} \end{array}$$

Precision (P) indicates the proportion of correctly identified targets among all samples identified as that target. Recall (R) represents the proportion of correctly identified targets among all actually existing targets of that class. Average Precision (AP) evaluates model performance by integrating both precision and recall, specifically by computing the area under the precision-recall curve at various thresholds. Mean Average Precision (mAP) means the average of the AP values across all classes, serving as a crucial metric for comprehensively assessing model performance in multi-class tasks. A higher mAP suggests better detection performance across multiple categories. In this study, *n* signifies the total quantity of whitefly adult and late-instar nymph classes, where *n* = 2. Additionally, to provide a more direct assessment of the model’s counting performance, the Root Mean Square Error (RMSE) was adopted as a supplementary metric. RMSE quantifies the average magnitude of the discrepancy between the predicted counts and the true counts, offering a clear measure of the model’s counting accuracy. The formula for RMSE is given by (Equation 11), where $y_{i}^{pred}$ and $y_{i}^{gt}$ represent the predicted and ground truth counts for the *i*-th image, respectively, and *N* is the total number of images in the dataset.


(11) $$\begin{array}{c} RMSE=\sqrt{\frac{\sum_{i=1}^{N}{\left(y_{i}^{pred}-y_{i}^{gt}\right)}^{2}}{N}} \end{array}$$

### 2.6 Intelligent monitoring and survey system for whitefly

In pursuit of automation, precision, and digital management of whitefly surveys, an intelligent survey system was developed for whitefly monitoring and surveying. This system uses a Browser/Server architecture that supports mobile applications—all established on a front-end/back-end separation design. The front-end interaction layer is developed via Vue for the web interface and Android for the mobile app, enabling functions like batch image uploads, visualization of detection results, and statistical analysis of detection data. The back-end service layer, built with the SpringBoot framework, is responsible for handling requests from both the web and mobile fronts, calling model inference interfaces, and performing data analysis. For data storage, MySQL is applied for model detection results, whereas Object Storage Service (OSS) saves original pest images and result images. Finally, the algorithm model service layer is developed based on the Flask framework to provide an automated whitefly counting interface, and is deployed to a dedicated algorithm server by virtue of Docker containerization technology.

## 3 Results

### 3.1 Comparison of foreground leaf segmentation models

To assess the performance of the Mask2Former-Leaf for foreground primary leaf segmentation, comparative experiments were conducted against mainstream semantic segmentation models, such as Fast-SCNN (Poudel et al., 2019), PSPNet (Zhao et al., 2017), DPT (Ranftl et al., 2021), Mask2Former (Cheng et al., 2022) and DeepLabv3+ (Chen et al., 2018). All models were trained and tested under identical experimental conditions. Specifically, Mask2Former was implemented with a ResNet-18 backbone, while DeepLabv3+ and PSPNet utilized a ResNetV1c-18 backbone. Fast-SCNN was implemented with its native customized backbone, and DPT employed a Vision Transformer backbone. As summarized by **Table 2**, the Mask2Former-Leaf model attained the best performance, with mIoU of 97.34%, mFscores of 98.65% and PA of 98.69%, respectively. This remarkably outperforms the other models, affirming its effectiveness in foreground primary leaf segmentation.

**Table 2:** Model comparison experiment for foreground leaf segmentation task.

Model	mIoU (%)	mFscore (%)	PA (%)	FPS
Fast-SCNN	77.51	87.33	87.60	4.01
PSPNet	83.29	90.89	90.23	5.85
DeepLabv3+	87.38	93.26	93.39	3.70
DPT	90.74	95.14	95.17	1.32
Mask2Former	93.23	96.50	96.57	2.14
Mask2Former-Leaf	97.34	98.65	98.69	1.88

### 3.2 Ablation experiment of the detection model

In order to ascertain the effectiveness of the three improved strategies in the YOLOv11-Whitefly model, this paper tested each of them using the same test set and compared the Precision (P), Recall (R), Average Precision (AP), and the mAP_50_. **Table 3** summarizes the results of this ablation study, where “√” implies the applied improvement strategy; “Adult” refers to whitefly adults; and “Nymph” means whitefly late-instar nymphs.

**Table 3:** Performance of three improvement strategies in the YOLOv11-Whitefly model for whitefly detection.

SAHI	DyCM-C3K2	MCRFPN	Precision (%)		Recall (%)		AP(%)		mAP_50_ (%)
			Adult	Nymph	Adult	Nymph	Adult	Nymph	
–	–	–	83.2	39.9	55.6	51.3	70.7	42.8	56.8
✓	–	–	92.7	75.1	91.6	71.3	97.2	80.2	88.7
✓	✓	–	92.9	76.6	93.7	75.6	97.4	84.0	90.7
✓	–	✓	92.2	75.2	93.5	77.1	97.1	83.5	90.3
✓	✓	✓	92.2	76.3	94.8	77.6	97.8	85.4	91.6

As listed in **Table 3**, the original YOLOv11s model performed poorly in detecting whiteflies in high-resolution images, with recall rates of just 55.6% for adults and 51.3% for late-instar nymphs, and an mAP_50_ of 56.8%. While SAHI is a general-purpose, architecture-agnostic strategy, it serves as an indispensable baseline for our high-resolution imagery. Integrating SAHI into the YOLOv11s model prominently upgraded performance, with recall rates for adults and late-instar nymphs jumped to 91.6% and 71.3%, respectively, while precision ascended to 92.7% and 75.1%. The AP for adults and late-instar nymphs rose to 97.2% and 80.2%, arousing an overall mAP_50_ of 88.7%. This demonstrates that, by slicing large images into sub-images, SAHI augmented the pixel proportion and morphological features of whiteflies, allowing the model to grasp richer feature representations during both training and inference. To further demonstrate the independent contribution and synergistic effect of our internal modules, we conducted additional experiments. Introducing DyCM-Conv into the backbone network to underpin the feature extraction module further boosted performance, particularly for the smaller late-instar nymphs. The late-instar nymph recall improved to 75.6%; precision grew by 1.5%; and AP rose to 84.0%—these results confirm that DyCM-Conv dynamically generates convolution kernels that adapt to input context, and this enhances feature extraction and captures long-range dependencies, so that small object detection can be fostered. Concurrently, replacing YOLOv11’s original neck network with the MCRFPN also demonstrated a significant performance boost, intensifying the average precision for whitefly adults and late-instar nymphs to 97.1% and 83.5%, respectively, spawning an mAP of 90.3%. This experiment corroborates that MCRFPN’s multi-scale feature fusion of superficial and deep features strengthens feature information interaction, and enhances the model’s ability to detect whiteflies of varying scales on different crop leaves. The full integration of all three strategies yielded the highest performance, achieving an mAP_50_ of 91.6%.

### 3.3 Comparison of different detection models

In this paper, to evaluate the detection performance of the YOLOv11-Whitefly, comparative experiments were carried out against several prominent detection models, which involved the one-stage models YOLOv10 (Wang et al., 2024) and RTMDet (Lyu et al., 2022), the two-stage model Cascade R-CNN (Cai and Vasconcelos, 2018), and the Transformer-based end-to-end object detection models DETR (Carion et al., 2020) and DINO (Zhang et al., 2022). Specifically, we chose ResNet-50 as the backbone for Cascade R-CNN, DETR, and DINO, while YOLOv10 and RTMDet utilized their native backbone architectures. For a fair comparison, all models were trained and tested on identical datasets using the same parameter settings. Given the difficulty of detecting tiny targets in our dataset, all models were also implemented with SAHI. The results of this evaluation are exhibited in **Table 4**.

**Table 4:** Performance comparison between YOLOv11-Whitefly and other detection models.

Models	Precision (%)	Recall (%)	mAP_50_ (%)	#Param (M)	GFLOPs	FPS
RTMDet-l	79.84	80.16	86.15	32.81	49.3	55.1
YOLOv10s	79.60	81.59	86.92	8.07	24.8	72.33
Cascade RCNN	74.30	78.01	81.25	69.16	239.0	15.42
DETR	75.61	80.13	83.04	41.58	86.0	35.7
DINO	76.08	78.68	82.68	47.70	269.0	11.25
YOLOv11-Whitefly(Ours)	84.25	86.20	91.60	23.84	56.8	43.16

**Figure 9:**
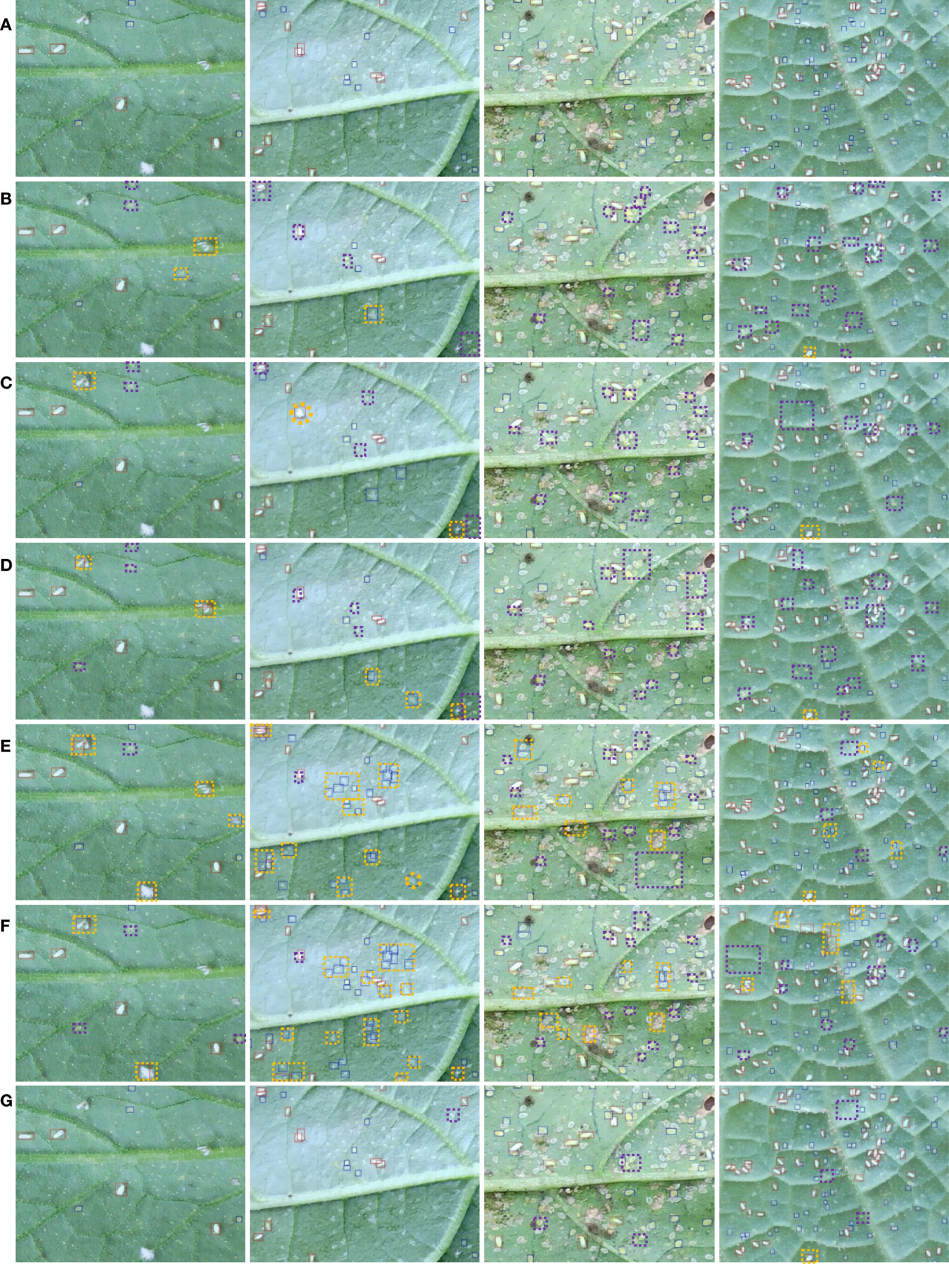
Examples of whitefly detection results on crop leaves using different models. **(A)** Manual annotation; **(B)** RTMDet; **(C)** YOLOv10s; **(D)** Cascade-RCNN; **(E)** DETR; **(F)** DINO; **(G)** YOLOv11-Whitefly (the proposed); Red and blue boxes indicate whitefly adults and late-instar nymphs, respectively; Purple and orange dashed boxes designate model misses and misdetects, respectively.

It can be seen from **Table 4** that the YOLOv11-Whitefly model attained the highest mAP_50_, surpassing RTMDet, YOLOv10s, Cascade R-CNN, DETR, and DINO by 5.45%, 4.68%, 10.35%, 8.56%, and 8.92%, respectively. For a more intuitive comparison, **Figure 9** displays localized magnified views of the whitefly detection results on various crop leaves generated by the aforementioned models. As shown in **Figure 9B-D**), traditional single-stage and two-stage detection models exhibit subpar performance in detecting whitefly late-instar nymphs, instigating noticeable missed detection. Concurrently, **Figure 9E, F** reflect that Transformer-based end-to-end object detection models generate numerous redundant prediction boxes, provoking a pronounced elevation in false positive rates. In contrast, the YOLOv11-Whitefly model proposed in this paper demonstrates superior robustness and stronger detection capabilities in intricate backgrounds, and lessens both missed detection and false positives.

To provide a more practical evaluation of the models’ final counting performance in real-world applications, we used the aforementioned detection models to count whiteflies on the original large images from the test set. For this experiment, all models were inferred with a confidence threshold of 0.3 and an IoU threshold of 0.5, and the SAHI method was applied to all predictions. The RMSE was calculated by comparing the results against the ground truth annotations. The RMSE for RTMDet, YOLOv10s, Cascade R-CNN, DETR, and DINO were 7.32, 7.07, 12.58, 9.74, and 10.51, respectively. Our model demonstrated superior counting accuracy, achieving the lowest overall RMSE of 4.49. A more granular analysis reveals that this high precision holds for both adult and late-instar nymph stages, achieving an RMSE of 3.99 for adult whiteflies and 1.39 for late-instar nymphs. This substantiates that our method’s high detection performance directly translates to a minimal counting error, which is a critical advantage for practical pest population surveys.

### 3.4 YOLOv11-whitefly model generalization experiment

To assess the cross-crop generalization capability of the YOLOv11-Whitefly model, a leave-out crop strategy was employed. Specifically, for this experiment, the model was trained exclusively on whitefly images from pepper, eggplant, and cucumber, ensuring that all images of cotton and tomato were strictly excluded from the training set. Subsequently, the model was evaluated on a complete test set comprising all five crops, with the test results summarized in **Table 5**. The model performed exceptionally on the crops it was trained on, and the mAP_50_ scores were 92.4% for pepper, 90.5% for eggplant, and 93.2% for cucumber. Crucially, the model also demonstrated a commendable detection ability on cotton and tomato, which was completely unexposed during the training stage. The model achieved 86.1% and 88.7% mAP_50_ on cotton and tomato crops, respectively. These results substantiate that the YOLOv11-Whitefly detection model is able to transfer its learning, and reliably detect whiteflies on unknown crop leaves, showcasing advantageous performance for cross-crop generalization.

**Table 5:** Performance of the YOLOv11-Whitefly model for detecting leaf whitefly in five different crops.

Metric	Pepper		Eggplant		Cucumber		Cotton		Tomato	
	Adult	Nymph	Adult	Nymph	Adult	Nymph	Adult	Nymph	Adult	Nymph
Precision(%)	93.4	76.9	91.9	78.5	90.5	80.4	97.0	73.0	90.3	80.9
Recall(%)	92.0	82.0	94.1	70.9	95.7	82.6	87.7	70.4	92.0	75.2
AP_50_(%)	97.2	87.6	97.8	83.2	97.1	89.3	97.7	74.5	96.5	80.9
mAP_50_(%)	92.4		90.5		93.2		86.1		88.7	

### 3.5 Visualization of the intelligent survey system for whitefly

**Figure 10:**
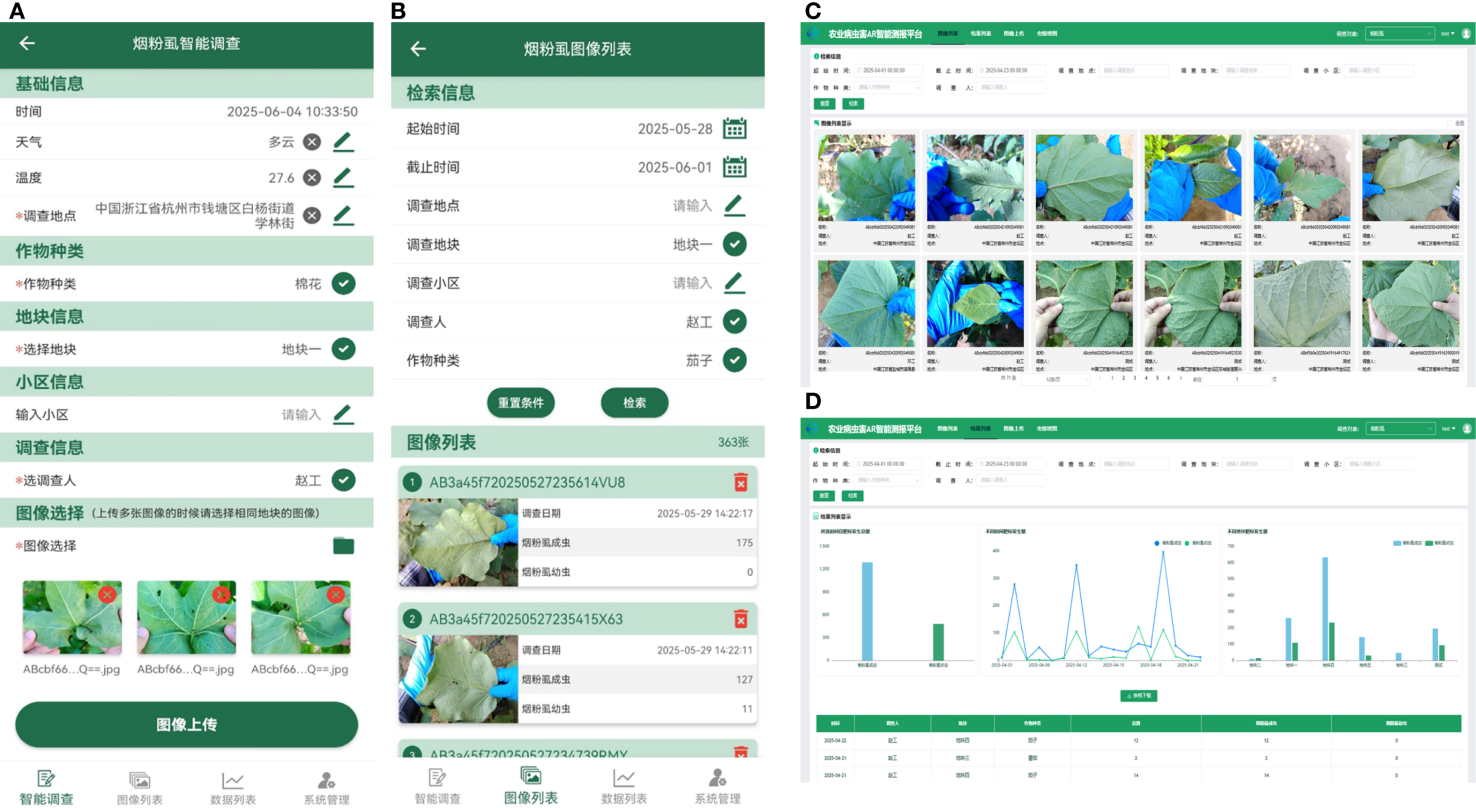
Interfaces of intelligent survey system for whitefly. **(A)** Intelligent survey interface in APP; **(B)** Results display interface in APP; **(C)** Image query interface in Web; **(D)** Whitefly data analysis interface in Web.

The functionalities of the intelligent survey system for whitefly pests designed in this study are illustrated in **Figure 10**, which encompass batch image upload for detection, image list display, and data analysis. Users may upload whitefly images in batches via the Web or mobile APP client interface. A whitefly counting interface call is sent by the backend server to the algorithm model, which sequentially processes the images through the Mask2Former-Leaf and YOLOv11-Whitefly models. The results are then returned to the back-end to undergo data persistence and encapsulation before being sent back to the front-end for final display.

## 4 Discussion

Benefited from the broadly permeated adoption of high-resolution image acquisition devices, the surging advancements in deep learning algorithms, and the bolstered accessibility and cost-effectiveness of computational resources, this paper focuses on the task of whitefly survey on crop leaves in natural field environments. An automatic whitefly counting method is put forth based on a segmentation-then-detection approach, utilizing AR glasses to acquire images from five types of crop leaves. Hence, an appropriate intelligent survey system has been developed, delivering a smart, efficient, and precise solution for monitoring and surveying the agricultural pest.

High-quality image data underlies the deep learning algorithms. From fixed cameras (Gao et al., 2025), smartphones (Xu et al., 2024), to professional cameras (Li et al., 2022; Ciampi et al., 2023), and custom-built pole-mounted photographic devices (Du et al., 2022; Li et al., 2023), a variety of portable, high-resolution image acquisition devices have been prevailing across numerous fields such as agricultural pest and disease detection. However, these machines confront some drawbacks. While fixed cameras save labor, their monitoring capabilities are confined to specific areas. Handheld devices (e.g. smartphones and cameras) offer solutions for manual field surveys, but are often perplexed by factors like strong illumination, fluctuating resolutions, and the necessity for manual operation. In contrast, AR glasses have been successfully applied to agricultural pest and disease image acquisition in studies by (Sheng et al., 2024; Chen et al., 2025; Ye et al., 2025), thanks to their hands-free operation, real-time visual display, and voice control functionalities. Therefore, this paper opted for the SUPERHEXA monocular AR glasses from Superhexa Century Technology for image acquisition, which is designed with a 50-megapixel main camera and a 3000-nits peak brightness optical display, and supports rapid autofocus and voice-controlled photography. By wearing these AR glasses, surveyors can utilize both hands to gently turn over and flatten leaves, which ensures a clear and stable view of the whiteflies for subsequent image capture. They also enable surveyors to view and adjust the content in real-time on the optical display, and directly trigger image capture via voice commands, so that the operational efficiency can be considerably enhanced. The AR glasses selected in this study are distinguished by powerful image acquisition capabilities and high imaging quality, capable of fulfilling the demands for automated counting of tiny whiteflies. Nevertheless, their detection accuracy for late-instar nymphs still compels improvement, which necessitates future work involving the AR glasses equipped with higher-resolution cameras. Another flaw lies in the AR glasses’ relatively small optical screen which could be replaced by a larger, higher-resolution display to ensure easier capturing of high-quality images.

Given that the whitefly agricultural pest monitoring standard NY/T 2950-2016 (2016) requires the counting of pests on single leaves, yet the images garnered by AR glasses in natural field environments often contain more than one leaf, thus precise extraction of the foreground primary leaf from the image implies immense importance for accurate whitefly enumeration. In recent years, semantic segmentation methods have demonstrated prominent merits in the field of crop disease detection. For instance, (Wang et al., 2021) utilized DeepLabV3+ and U-Net models for segmenting diseased areas on cucumber leaves in complex backgrounds; (Zhang et al., 2023) proposed the Locally Reversible Transformer model for grape leaf disease segmentation in natural scenes; and (Ye et al., 2025) introduced the DeepLab-Leafminer model for segmenting leafminer damage regions. Inspired by these accomplishments, this paper harnesses the Mask2Former model combined with a mask post-processing optimizer to precisely segment the foreground primary leaf from whitefly images across various crop types in natural environments. This initial segmentation stage effectively attenuates interference from intricate backgrounds, and minimizes false positives arising from whiteflies not located on the primary leaf, so that a cleaner and more focused region for subsequent detection is provided.

In the domain of tiny agricultural pest detection, several advanced object detection models marking notable progress have been presented in existing research, as exemplified by YOLO-Pest (Xiang et al., 2023), DRB (Jiao et al., 2022), MDM (Wang et al., 2020), and Cascade-RCNN-PH (Sheng et al., 2024). To enable precise detection of tiny whitefly adults and late-instar nymphs in the foreground primary leaf, this paper introduces the DyCM-C3K2 module based on the YOLOv11 model, so as to construct the YOLOv11-Whitefly model by reinforcing the backbone network, substituting the original neck network with MCRFPN, and merging SAHI capabilities. Notwithstanding that the segmentation-then-detection method for whitefly counting realizes high-accuracy detection on various crop leaves, the sequential execution of two independent deep learning models inevitably deteriorates the model’s computational load and inference time. To address this, future efforts could be invested in a more comprehensive exploration of model lightweighting strategies. In addition to model pruning (Sun et al., 2023) and knowledge distillation (Hsieh et al., 2023), techniques such as model quantization could be employed to reduce the computational complexity and accelerate inference speed without compromising the high level of feature extraction and detection performance. Furthermore, a crucial step for improving throughput would be to deploy the model directly onto edge devices, such as the AR glasses themselves, thereby eliminating network latency and ensuring a more responsive system for on-site applications.

Despite the fruits harvested in whitefly detection in this study, a number of defects perpetuate. Our dataset reflects a class imbalance with adults being more prevalent than late-instar nymphs, which is a direct consequence of the inherent challenges of professional field data acquisition where pest populations naturally exhibit a non-uniform and imbalanced distribution. This also resulted in substantial variability in pest density, as reflected by high standard deviations and a wide range of counts, from low-density scenarios to high-density clusters. To mitigate these issues and further enhance model performance, a key focus for future endeavors will be on data expansion. This involves collecting a more balanced dataset with additional nymph-rich images and employing more systematic sampling protocols to ensure a more uniform representation of pest populations across various densities. Furthermore, we will incorporate images from a wider variety of host crops to improve the model’s generalization capabilities. While the present study focuses exclusively on whitefly (*Bemisia tabaci*), a dominant species in our data collection areas, agricultural practices indicate that greenhouse whitefly (*Trialeurodes vaporariorum*) is also an important pest that frequently co-occurs in other regions. The morphological similarity presents a significant challenge for machine vision-based discrimination. Accordingly, a crucial direction for future work is to develop a robust model capable of differentiating between these two species, which will substantially enhance the model’s practical utility in regions where they co-exist.

## 5 Conclusions

Aimed at efficiently surveying whitefly populations on crop leaves in natural field environments, this study proposes an automatic whitefly counting method based on AR glasses and a segmentation-then-detection model. Acquired by the voice-control of the surveyors wearing AR glasses, the images of whiteflies on the undersides of crop leaves are transmitted to a server that activates an automatic whitefly detection model to count the insect population. In pursuit of the minimal interference from non-primary leaf areas and intricate field backgrounds, Mask2Former-Leaf is engaged to segment the foreground primary leaf in the images. For the sake of accurately detecting tiny whitefly adults and late-instar nymphs in the images, SAHI was integrated into the YOLOv11 model, and enhanced by incorporating DyCM-C3K2 and MCRFPN. The final model attained an average precision of 84.25% and an average recall rate of 86.20% for whitefly detection, apart from an mAP_50_ of 91.60%. Furthermore, the Web/APP client of the intelligent survey system for whitefly monitoring and surveying can synchronously display whitefly images and their counts. Its data analysis features help surveyors understand whitefly occurrences and elaborate feasible prevention and control strategies. The proposed method presented can also be extended to forecast and survey cotton aphid, thrips and other agricultural pests in natural fields, thereby advancing the intelligent development of agricultural pest surveys.

## Data Availability

The raw data supporting the conclusions of this article will be made available by the authors, without undue reservation.
